# Use, misuse, and pitfalls of the drug challenge test in the diagnosis of the Brugada syndrome

**DOI:** 10.1093/eurheartj/ehad295

**Published:** 2023-06-22

**Authors:** Arthur A M Wilde, Ahmad S Amin, Hiroshi Morita, Rafik Tadros

**Affiliations:** Department of Clinical and Experimental Cardiology, Heart Center, Amsterdam Cardiovascular Sciences, Amsterdam UMC, University of Amsterdam, Room B2-256, Meibergdreef 9, Amsterdam 1105 AZ, The Netherlands; European Reference Network for rare, low-prevalence, or complex diseases of the heart (ERN GUARD-Heart), Amsterdam, The Netherlands; Department of Clinical and Experimental Cardiology, Heart Center, Amsterdam Cardiovascular Sciences, Amsterdam UMC, University of Amsterdam, Room B2-256, Meibergdreef 9, Amsterdam 1105 AZ, The Netherlands; European Reference Network for rare, low-prevalence, or complex diseases of the heart (ERN GUARD-Heart), Amsterdam, The Netherlands; Department of Cardiovascular Medicine, Okayama University Graduate School of Medicine, Dentistry and Pharmaceutical Sciences, Okayama, Japan; Department of Cardiovascular Therapeutics, Graduate School of Medicine, Dentistry and Pharmaceutical Sciences, Okayama University, Okayama, Japan; Cardiovascular Genetics Center, Montreal Heart Institute, Faculty of Medicine, Université de Montréal, Montreal, Québec, Canada

**Keywords:** Brugada syndrome, Sodium channel blocker, Drug challenge test, Risk stratification, Genetics

## Abstract

The diagnosis of Brugada syndrome (BrS) requires the presence of a coved (Type 1) ST segment elevation in the right precordial leads of the electrocardiogram (ECG). The dynamic nature of the ECG is well known, and in patients with suspected BrS but non-diagnostic ECG at baseline, a sodium channel blocker test (SCBT) is routinely used to unmask BrS. There is little doubt, however, that in asymptomatic patients, a drug-induced Brugada pattern is associated with a much better prognosis compared to a spontaneous Type 1 ECG. The SCBT is also increasingly used to delineate the arrhythmogenic substrate during ablation studies. In the absence of a “gold standard” for the diagnosis of BrS, sensitivity and specificity of the SCBT remain elusive. By studying patient groups with different underlying diseases, it has become clear that the specificity of the test may not be optimal. This review aims to discuss the pitfalls of the SCBT and provides some directions in whom and when to perform the test. It is concluded that because of the debated specificity and the overall very low risk for future events in asymptomatic individuals, patients should be properly selected and counseled before SCBT is performed and that SCBT should not be performed in asymptomatic patients with a Type 2 Brugada pattern and no family history of BrS or sudden death.

## Graphical Abstract

**Graphical Abstract:**
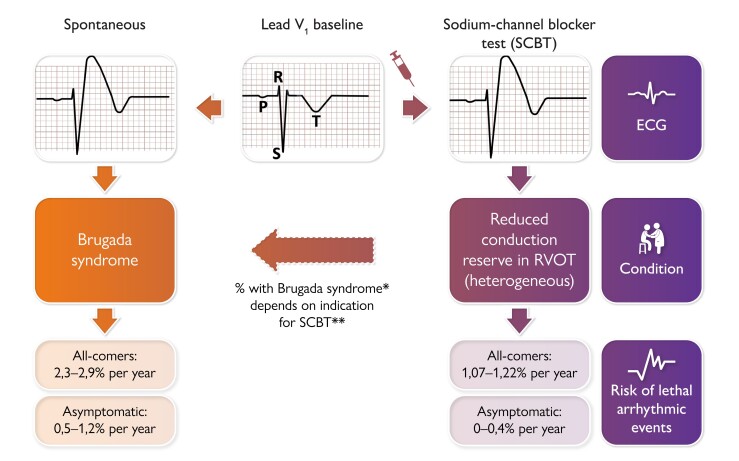
Use, misuse, and pitfalls of the drug challenge test in the diagnosis of the Brugada syndrome. *Diagnosis of Brugada syndrome is based on the modified Shanghai criteria (see *Table 2*). **Indications to perform SCBT depend on symptoms, family history, and baseline ECG. Before performing the test, patients should be informed about test specificity, risk of complications, and generally excellent prognosis if asymptomatic. All-comers: asymptomatic patients and patients with symptoms including ventricular fibrillation/aborted cardiac arrest. Lethal arrhythmic events: sudden cardiac death, ventricular fibrillation or sustained ventricular tachycardia, and appropriate implantable cardioverter-defibrillator interventions (i.e. shocks or antitachycardia pacing delivered for ventricular tachycardia/fibrillation). ECG, electrocardiogram; RVOT, right ventricular outflow tract; SCBT, sodium channel blocker test.

## Introduction

Brugada syndrome (BrS) is an inherited arrhythmia syndrome associated with an increased risk of ventricular arrhythmia (VA) and sudden cardiac death.^1,2^ The diagnosis requires the presence of a coved (Type 1) ST segment elevation in the right precordial leads of the electrocardiogram (ECG). A notorious feature of BrS that was highlighted shortly after the initial report of the syndrome is the dynamic nature of the ECG, whereby the resting ECG may often be normal or non-diagnostic in many patients at certain time points. Therefore, in patients with suspected BrS but non-diagnostic ECG at baseline, a sodium channel blocker test (SCBT) is routinely used to unmask BrS.

In the first consensus statements on BrS, published in 2002 and 2005, a positive SCBT by itself was regarded as insufficient for the diagnosis BrS, and additional criteria were required.^3,4^ This changed in 2013 when a drug-induced ECG alone was accepted as diagnostic.^5^ However, more recently (2016), the presence of additional criteria was again suggested in order to avoid overdiagnosis.^1^ Given major concerns on the specificity of the SCBT, some authors have even ironically wondered whether “everybody had BrS until proven otherwise.”^6^

This review aims to discuss the pitfalls of the SCBT. The struggle to establish the gold standard for the test, with, by definition, significant implications for the specificity and the sensitivity of the test is emphasized.

### Historical notes

Although reported earlier,^7,8^ BrS is named after the Spanish cardiologists Pedro and Josep Brugada who described the condition in 1992.^9^ A few years later, the effect of sodium channel blockers (SCBs) in suspected BrS was described in 1996 in just three patients.^10^ In this study, the effect of various pharmacological interventions, including procainamide (one patient) and disopyramide (two patients; “Class 1A” drugs) was studied. In all three patients, the baseline ECG was non-diagnostic, and after exposure to either drug, in addition to the appearance of ventricular extrasystoles, augmentation of the ST segment was observed, without actually reaching a full-blown Type 1 ECG in two out of three patients. A year later, Josep and Pedro Brugada described that in six BrS patients with a temporarily normal ECG, ajmaline and procainamide “unmask the described ECG pattern.”^11^ The first attempt to describe the sensitivity and specificity of the test was published in 2000.^12^

### Pathophysiology of the sodium channel blocker test

**Figure 1:**
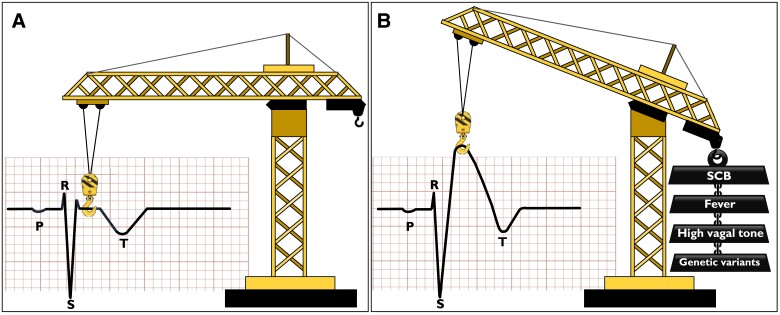
Schematic representation of the effects of genetic and environmental factors in Brugada syndrome. (*A*) Normal ST segment pattern in the right-precordial lead *V*_1_. (*B*) Acquired factors such as sodium channel blockers, fever, or increased vagal tonus or genetic factors act in a conjoint manner to reduce the depolarization reserve and delay electrical conduction in the right ventricular outflow tract, thereby leading to coved-type (Type 1) ST segment elevation in the right-precordial leads (here *V*_1_). SCB, sodium channel blocker.

The pathophysiology underlying the SCBT-induced augmentation of ST segment elevation in the right precordial leads is probably very much related to the mechanisms of the spontaneous Type 1 ECG (*Figure 1*). We firmly believe that the main determinant of the right precordial ST segment elevation is a profound conduction delay in the right ventricular outflow tract (RVOT) area associated with what is referred to as “reduced RVOT conduction reserve.”^2^ Such reduced conduction reserve is mediated by both genetically mediated reduction in sodium current^13^ and other genetic or acquired factors including fibrosis.^14^ In those individuals where the RVOT abnormalities do not lead to a spontaneous Type 1 ECG under given circumstances, SCBs may tip the balance to the degree of conduction delay or extend the area with conduction delay, needed for a Type 1 ECG. Indeed, epicardial mapping studies revealed that there is a consistent relation between the size of the area with abnormal fractionated low-voltage signals and the degree of ST elevation, and SCBs consistently enlarge that particular area with concomitant appearance of a Type 1 pattern.^15^

### Sensitivity and specificity of the sodium channel blocker test

In order to assess the sensitivity of a diagnostic test, a “gold standard” to which the test is compared is required. A challenging problem is that there actually is no absolute gold standard for BrS other than the Type I pattern, and previous studies have used patients with different characteristics as their gold standard. The most frequently used surrogate gold standard is carriership of a familial *SCN5A* variant. The initial results using this approach were promising with a positive SCBT in all tested individuals with a pathogenic variant (*n* = 11, using procainamide, flecainide, and ajmaline in all) and a negative response in their genotype-negative family members (*n* = 8).^12^ However, over time, quantification of the sensitivity by using *SCN5A* carriership status as the gold standard reveals it to be 76%–80% for ajmaline^16–18^ and 77% for flecainide.^19^

Patients with an intermittent spontaneous Type 1 are another patient group often used as a gold standard. In the same early study by Brugada and Brugada, 34 patients with intermittent Type 1 ECG all responded positive to a SCBT using ajmaline, flecainide, or procainamide.^12^ Later studies clearly demonstrated that ajmaline is more sensitive than flecainide (100% vs. 68%).^20^ Procainamide is generally considered even less sensitive, but a systematic comparison of procainamide has not been performed to our knowledge.^21^

A third option is performing the SCBT in obligate transmitters of a known or unknown variant. This, rather unique, elegant approach was used by Therasse *et al*.^18^ An obligate carrier is a person connecting two affected relatives in a pedigree. This method, employed in both families with (*n* = 20) and without (*n* = 22) a pathogenic variant, revealed a sensitivity for ajmaline (*n* = 37) and flecainide (*n* = 13) testing of 100% and 77%, respectively. The method may overestimate sensitivity as a test is probably not performed in first-degree relatives of an individual with a negative test.^22^ Furthermore, this method assumes an autosomal dominant transmission, whereas BrS is known to have a complex inheritance.^23,24^ However, based on all these studies, it can probably be concluded that ajmaline can be used to safely rule out BrS, but flecainide results should be interpreted more carefully due to its lower sensitivity.

Ajmaline is not available in many countries, including Canada, the USA, and Japan. In Canada and the USA, intravenous procainamide infusion is used as a SCBT in suspected BrS.^25^ As mentioned, the sensitivity of procainamide has not been defined, and direct comparison with ajmaline has not been done in the same patients. In countries where no intravenous ajmaline, flecainide nor procainamide is available (i.e. Latin American countries), oral flecainide has been used (without data on sensitivity and specificity).^26^ A recent study from Canada and the UK compared the rate of positive SCBT in similar patient populations (not identical).^27^ In multivariable analyses, use of ajmaline (compared to procainamide) was associated with an ∼9-fold increased odds for a positive SCBT challenge, highlighting the markedly increased sensitivity of ajmaline vs. procainamide. In Japan, procainamide and disopyramide were used for SCBT in early studies.^10,28^ Pilsicainide is a pure SCB,^29^ which is available in Japan; in oral form, it was used from 1991, and injection of the drug was released in 2000. The first study using pilsicainide was published in 1999,^30^ and pilsicainide has since been widely used in Japan,^31,32^ Positive tests of pilsicainide were reported in 34%–83% of patients with suspected BrS,^33,34^ but there has been no study on the test sensitivity and specificity of pilsicainide challenge in patients with pathogenic variants. In our experience, the sensitivity of pilsicainide test in patients with *SCN5A* mutation is 95.5% (unpublished data). False-positive and false-negative results of pilsicainide tests should exist, but their incidences have not been reported.

Although the sensitivity of ajmaline (and pilsicainide) is sufficiently high to be used in ruling out BrS in those with suspected diagnosis but no documentation of a Type 1 ECG at baseline, the low specificity in those without suspected BrS is a major concern and may result in overdiagnosis. In the last years, many groups have reported alarmingly high rates of positive SCBT using ajmaline in patient populations with no suspected BrS (*Table 1*). For instance, positive ajmaline tests have been reported in other malignant arrhythmogenic diseases including ∼16% of patients with arrhythmogenic right ventricular cardiomyopathy^36,37^ and ∼18% of patients with myotonic dystrophy^38,39^ but also generally benign conditions such as in ∼27% of patients with atrioventricular (AV) nodal reentrant tachycardia (AVNRT)^35^ and ∼16% of patients with AV accessory pathways.^40^ Even more concerning are studies by Hasdemir and colleagues reporting that SCBT with ajmaline may be positive in nearly 5% of a control population.^35,40^ In addition, we recently showed that in 8% of family members of sudden death fatalities who were diagnosed as BrS based on ajmaline test, the diagnosis was ultimately found to represent a false-positive diagnosis, emphasizing the potential for confounding possibly false-positive ajmaline responses in this population (*see further*).^41^ Studies on specificity of SCBT using other less potent SCBs such as procainamide are lacking, and as such, the rate of “false-positive” tests remains unknown but expected to be very low. This striking lack of specificity of ajmaline in patients with no suspected BrS was the main reason why in subsequent consensus statements a positive SCBT alone is now no longer regarded as diagnostic for BrS. As stated in the modified Shanghai criteria, other clinical features are required to diagnose BrS in the absence of a spontaneous Type 1 ECG (*Table 2*). Pending data on specificity of other SCBs, this diagnostic recommendation extends beyond ajmaline and also applies to procainamide and pilsicainide.

**Table 1:** Patients with conditions in whom a SCBT was performed

Condition	No. of patients	Positive SCBT	No. of controls	Positive SCBT	Reference
ARVC	55	16%			36
ARVC	106	16%			37
AVNRT	96	26 (27.1%)	66	3 (4.5%)	35
Atrioventricular accessory pathway	124	20 (16.1%)	84^a^	4 (4.8%)	40
Chagas disease	101	7 (6.9%)	46	0 (0%)	119
Myotonic dystrophy	44	18%			38
Myotonic dystrophy.	12	25%			39
**Spontaneous Type 1 ECG**					
Fever	24 5 7	Ajmaline: 80.0% Flecainide: 79.2% Pilsicainide: 85.7%			120
SBMA	144	17 (11.8%)			121
SBMA	30	2 (6.6%)			122
ARVC	114	5 (4%)			123
**Induced Type 1 ECG by specific condition**					
Cyclic antidepressants overdose	98	15 (15.3%)			124
Antiepileptic drug	120	15 (12.5%)			125
Hypercalcemia	28	6 (21%)			126
Hyperkalemia	59	15 (25%)			127
Propofol	67	6 (9%)			128

In this study, 66 controls were taken from the previous 2015 study.^35^

AVNRT, AV-nodal reentry tachycardia; SBMA, spinal and bulbar muscular atrophy.

**Table 2:** Modified Shanghai scoring system for the diagnosis of BrS

Criteria	Points
I. ECG (at least one ECG criterion is required for diagnosis) Only award points once for highest score within this category	
Spontaneous Type 1 Brugada ECG pattern at nominal or high leads	3.5
Fever-induced Type 1 Brugada ECG pattern at nominal or high leads	3
Sodium channel blocker-induced Brugada Type I ECG pattern at nominal or high leads	2
II. Clinical history Only award points once for highest score within this category	
Unexplained cardiac arrest or documented VF/polymorphic VT	3
Nocturnal agonal respirations	2
Suspected arrhythmic syncope	2
Syncope of unclear mechanism/unclear etiology	1
Atrial fibrillation/flutter in patients <30 years without alternative etiology	0.5
II. Family history (first or second degree relative) Only award points once for highest score within this category	
Definite BrS	2
Suspicious SCD (fever, nocturnal, and Brugada-aggravating drugs)	1
Unexplained SCD <45 years with negative autopsy	0.5
IV. Genetic test result	
Pathogenic or likely pathogenic genetic variant in *SCN5A*	0.5

Score: >3.5 points required for probable/definite Brugada syndrome (BrS); 2–3 points for possible BrS; <2 points is considered non-diagnostic.

### Safety of the sodium channel blocker test

Ventricular arrhythmias can occur during SCBT and are reported mostly in patients with a positive test but occasionally also in those who did not reach Type 1 ECG (maybe due to premature termination of the SCBT due to VA).^16,42^ Sodium channel blocker test-induced VA include premature ventricular contractions (PVCs), non-sustained or sustained, monomorphic or polymorphic; ventricular tachycardia (VT), sometimes requiring isoproterenol infusion or external defibrillation for termination; and ventricular fibrillation (VF).^34,43–45^ Anecdotally, occurrence of refractory VF during SCBT has been reported, requiring prolonged resuscitation and/or venoarterial extracorporeal membrane oxygenator (ECMO) placement.^45–47^ The incidence of malignant VA (i.e. VF, sustained VT, or VT requiring intervention) during SCBT ranged from 0% to 10.7% (mean ∼1.4%).^9,12,16,19–21,34,43–45,48–58^ Because of the low rate of events in most studies, heterogeneous patient cohorts, different types of SCB used, and the lack of a systematic approach with regard to genotyping, it remains difficult to identify factors that unambiguously predict the occurrence of SCB-induced VA. However, the available data suggest that the risk for SCB-induced VA is higher in patients with spontaneous Type 1 Brugada ECG pattern and/or prolonged conduction intervals at baseline and in patients with a pathogenic variant in *SCN5A*.^34,44,45,48^ Interestingly, each of these factors has been associated with larger degree of cardiac sodium current reduction, suggesting that SCB-induced VA may depend on the magnitude of sodium current inhibition during SCB infusion. The role of *SCN5A* mutations as a risk factor for VA during SCBT has been further supported by a report of ajmaline-induced VA in multiple members of a large family with truncating *SCN5A* variants and the identification of family history of VA during SCBT as a predictor of SCB-induced VA.^59,60^

In our study including up to 1368 patients who underwent testing, the rate of ajmaline-induced ventricular ectopy was 4%, including PVCs and non-sustained VT.^61^ There was no case of sustained VT/VF. Notably, ajmaline infusion was interrupted at the appearance of ventricular ectopy and appearance of a Type 1 pattern, and/or if important, QRS prolongation was observed. This may have resulted in a lower incidence of significant adverse arrhythmic events than expected from other cohorts. The presence of a pathogenic *SCN5A* variant was a predictor of ajmaline-induced ventricular ectopy. In a secondary non-pre-specified analysis, a higher burden of common variants associated with BrS was also significantly associated with ajmaline-induced ventricular ectopy.^61^

Younger age at the time of SCBT and sinus node dysfunction (SND) have also been associated with the occurrence of VA during SCBT.^45^ Currently, there is no rationale for the increased rate of SCBT-induced VA in younger individuals, but, hypothetically, this could be explained by a higher frequency of *SCN5A* mutations or an increased vagal tonus. On the other hand, it is important to note that no study has found evidence that pediatric patients (<18 years) are at higher risk of VA during SCBT. Whether the presence of SND is an independent predictor of VA during SCBT (and worse prognosis in adult BrS patients) remains debatable, as the reported numbers of patients with SND and SCB-induced VA or adverse events during follow-up are limited and often involve children and/or patients with a *SCN5A* mutation.^45,62^

Usually, the administration of a SCB during a provocation test is immediately terminated when a diagnostic Brugada Type 1 ECG develops or when PVCs, QRS prolongation (by ≥30% of the baseline value), or second- or third-degree AV block occurs. Accordingly, severe VAs have been reported in a few cases where SCB infusion was not stopped while a diagnostic Type 1 ECG was reached or PVCs had occurred.^42,43^ It is not clear whether not terminating SCB infusion when QRS prolongation ≥30% is reached increases the risk of VA. Batchvarov *et al.* not only did not find an association between QRS widening ≥30% and VA during ajmaline testing but also showed that in 40% of the positive tests, the diagnostic Type 1 ECG appeared only after the QRS had widened >30% of the baseline values.^50^ So, whether the extent of QRS widening during SCBT should be adjusted for baseline QRS intervals to be appropriately used as a termination criterion or whether QRS widening ≥30% is only a risk factor for SCB-induced VA in patients with signs of intraventricular conduction delay at baseline remains to be investigated.

Regardless of the risk of VA, one should keep in mind that SCB infusion may also result in transient complete AV block (with ventricular asystole), particularly in older patients or those with a loss-of-function mutation in *SCN5A* with prolonged conduction intervals at baseline. In addition, special attention may be required with regard to body weight, as we are aware of (unpublished) cases of VF storm after ajmaline infusion in persons with overweight who received 1 mg/kg, reaching a total amount of more than 100 mg ajmaline.

As life-threatening VAs during SCBT are not uncommon, it is extremely important that the test is performed by experienced healthcare providers under strict medical surveillance, in an environment with direct access to basic life support facilities. Monitoring after the SCBT is required until the ECG returns to its baseline morphology. The treatment of VA during SCBT is challenging and may require isoproterenol and external (or internal) defibrillator shocks. If not successful, an ECMO placement should be considered. Transient complete AV block may require temporary pacemaker therapy.

Recently, infusion of a SCB has been increasingly used to uncover or aggravate the abnormal arrhythmogenic substrate in the RVOT, or sometimes even beyond the RVOT, for the purpose of catheter ablation in BrS patients.^15^ Low-voltage, prolonged, and fractionated electrograms (EGMs) as possible targets for substrate ablation may be absent or minimal at baseline. In such circumstances, intravenous infusion of a SCB, often ajmaline (in >75% of all cases) using a protocol similar to SCBT (e.g. 1 mg/kg in 5 min), has been shown to effectively reveal or expand such substrate regions for ablation. Unfortunately, ablation studies in BrS often do not report the rates of VA occurrence during SCB infusion. However, it is expected that these rates are comparable with those during diagnostic SCBT and perhaps even higher as more severely affected BrS patients may undergo such procedures.

Beyond the abovementioned arrhythmic complications of SCBT, ajmaline infusion has also been associated with cholestatic liver injury.^63,64^ The prevalence of liver toxicity remains unknown, but clinically detectable toxicity is thought to be rare.

### Factors impacting on the sodium channel blocker test

Fundamentally, the SCBT measures the sensitivity of an individual to sodium channel blockade at a given time point. Although such sensitivity to sodium channel blockade is a quantitative trait, for diagnostic purposes in BrS, the test result is defined as either “positive” (if reaching criteria for a type I BrS ECG pattern, i.e. coved-type with ≥2 mm ST elevation in *V*_1_ or *V*_2_), “negative” (in the absence of a diagnostic Type I pattern at the target drug dose), or “inconclusive” (in the absence of a diagnostic Type I pattern in a test that was prematurely terminated before the target sodium channel dose is reached). The test result depends on factors related to (i) the test characteristics, (ii) intrinsic non-modifiable patient characteristics, and (iii) exogenous factors impacting SCB sensitivity.

#### Test characteristics

As previously mentioned, the specific SCB used is a major determinant of test result. Although direct comparisons are largely missing, it is known that procainamide is less sensitive (and perhaps more specific) than ajmaline for eliciting the Type I pattern.^27^ Flecainide is also less sensitive than ajmaline.^18,60^ Sodium channel blocker dosing is another determinant of test result. Not surprisingly, higher ajmaline dosing (beyond 1 mg/kg) increases the rate of Type 1 pattern but has been shown to result in BrS overdiagnosis and should therefore be avoided.^41^ The drugs used in SCBT and recommended target doses are listed in *Table 3*. A last test characteristic which influences test result is the recording of *V*_1_–*V*_2_ at higher intercostal spaces (second and third intercostal spaces). High lead recording increases the sensitivity of SCBT, but the effect on specificity has not been directly addressed.^65^

**Table 3:** Agents and dosing for sodium channel blocker tests

Drug	Administration	Dose	Infusion duration or rate
Ajmaline	Intravenous	1 mg/kg	5–10 min or 10 mg/minute
Flecainide	Intravenous	2 mg/kg, up to 150 mg	10 min
Flecainide	Oral	200–300 mg	NA
Procainamide	Intravenous	10–15 mg/kg; up to 1000 mg	10–20 min or 100 mg/min
Pilsicainide	Intravenous	1 mg/kg	5–10 min or 10 mg/minute

#### Intrinsic patient characteristics

In light of the heritability of BrS, the individual genetic profile plays an important role in SCBT result. Patients carrying a pathogenic *SCN5A* variant have a higher likelihood of having a positive SCBT.^57,61^ Beyond rare pathogenic *SCN5A* variants, common variants previously associated with BrS have also been found to be associated with SCBT-induced Type I pattern. Specifically, a polygenic risk score comprised of three common variants at the *SCN5A*, *SCN10A*, and *HEY2* loci (PRS_BrS_) was a strong independent predictor of ajmaline-induced Type 1 pattern with clinically relevant discriminative ability,^61^ suggesting that PRS_BrS_ may become a clinical tool to identify patients at risk of SCBT-induced Type 1 pattern. Interestingly, although males are known to be at higher risk for BrS overall, sex is not associated with SCBT result in different cohorts using different drugs.^27,60,61^ Age is another determinant of SCBT result in univariable analyses, with older patients at significantly at higher odds of having a positive SCBT.^60,61^ Nonetheless, age is not an independent predictor in multivariable analyses. Last, features on the baseline ECG are also associated with SCBT result, with larger QRS durations and presence of a Type 2 BrS ECG being associated with a positive test,^60,61^ as are specific QRS morphological features.^60^

#### Exogenous factors

**Figure 2:**
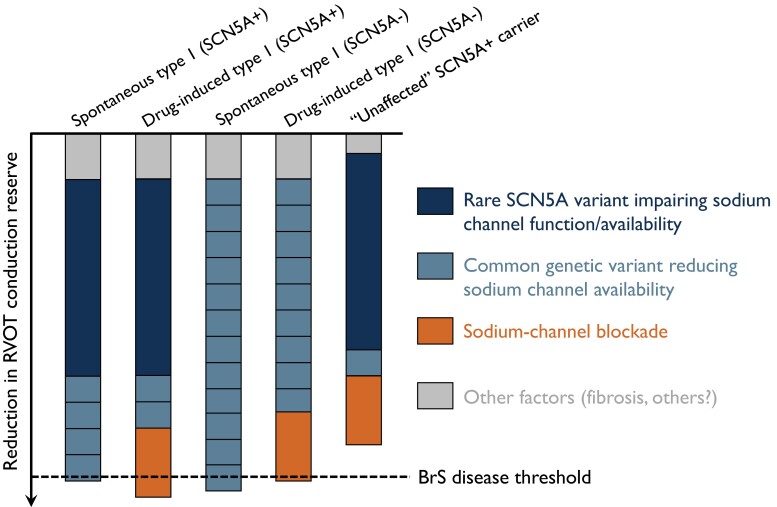
Schematic representation of genetic and non-genetic determinants of Brugada syndrome. Shown are five distinct hypothetical patients with BrS and/or pathogenic *SCN5A* variants. The central mechanism underlying Brugada syndrome is a reduction in right ventricular outflow tract conduction reserve, mediated by any combination of the following: rare pathogenic *SCN5A* variant reducing sodium channel function, common genetic variants affecting sodium channel availability, exogenous sodium channel blockade, and other factors including fibrosis in the right ventricular outflow tract. The sodium channel blocker test consists in unmasking the Brugada syndrome phenotype in individual with underlying Brugada syndrome. The magnitude of each contributor to Brugada syndrome is schematically represented and does not necessarily reflect relative contributions which have not yet been quantified. BrS, Brugada syndrome; RVOT, right ventricular outflow tract.

Exogenous factors affecting SCBT have not been systematically studied. Intuitively, co-administration of medication which blocks the cardiac sodium channel is expected to falsely increase risk for a positive test, and it is best to withhold such medication prior to testing. A list of such medications can be found on www.brugadadrugs.org.^66^ Hyperkalemia can also reduce cardiac sodium channel availability, and, if suspected, it should be verified and corrected particularly if severe before SCBT (*Figure 2*).

### Prognosis associated with a positive test outcome

Generally, patients with drug-induced Type 1 ECG have a better prognosis compared to patients with spontaneous Type 1 ECG. Patients with non-Type 1 ECG in whom a SCB does not convert to Type 1 ECG have a more benign clinical course than that in patients with drug-induced Type 1 ECG.^34,60,67,68^

The presence of symptoms also impacts on the prognosis of patients in whom the SCBT is positive. In all-comers (i.e. asymptomatic patients and patients with symptoms including VF/aborted cardiac arrest), the incidence of lethal arrhythmic events is reported to be 2.3%–2.87%/year in the presence of a spontaneous Type 1 ECG and 1.07%–1.22%/year in the presence of a drug-induced Type 1 ECG.^34,56,68–75^ However, other studies failed to show a difference in the prognosis of patients with spontaneous Type 1 ECG and that of patients with drug-induced Type 1 ECG.^72,76–79^ The difference in results between studies might be due to different follow-up durations, different ratios of patients having spontaneous or drug-induced Type 1 ECG, and different incidences of previous VF or arrhythmogenic syncope. Among patients with drug-induced Type 1 ECG, presence of symptoms, VF-induced by programmed electrical stimulation, proband status, and atrial fibrillation were reported as risk factors for VF.^45,80^

**Figure 3:**
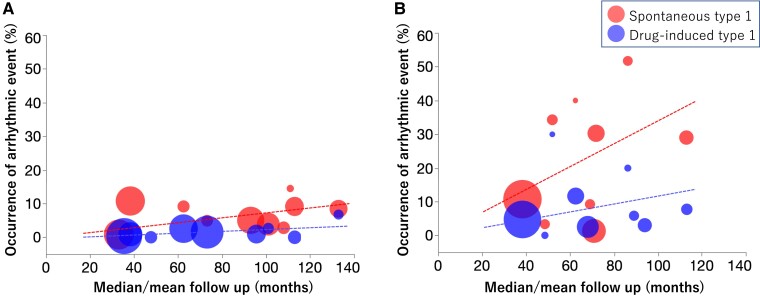
Bubble plot. *(A*) Prognosis of asymptomatic patients. Red circles show occurrence of arrhythmic event in asymptomatic patients with spontaneous Type 1 electrocardiogram, and blue circles show that in asymptomatic patients without spontaneous Type 1 electrocardiogram. Bubble size represents the number of patients. Dotted lines show approximate line of the event ratio of each group. Patients with drug-induced Type 1 electrocardiogram had better prognosis (0.29%/year) than patients with spontaneous Type 1 electrocardiogram (0.88%/1year).^34,56,69,76,81–86^ The average event ratio was calculated from a formula of approximate line (asymptomatic spontaneous Type 1: *y* = 0.074 * 12 * *x*, asymptomatic drug-induced Type 1: *y* = 0.024 * 12 * *x*, *x* = follow-up months). *(B*) Prognosis of symptomatic patients including syncope and ventricular fibrillation/aborted cardiac arrest. Red circles represent symptomatic patients with spontaneous Type 1 electrocardiogram and blue circles show symptomatic patients without spontaneous Type 1 electrocardiogram. Patients with spontaneous Type 1 electrocardiogram had worse prognosis (4.08%/year) than patients with drug-induced Type 1 electrocardiogram (1.34%/year).^34,56,69,76,81,83,87^ The average event ratio was calculated from a formula of approximate line (symptomatic spontaneous Type 1: *y* = 0.340 * 12 * *x*, symptomatic drug-induced Type 1: *y* = 0.116 * 12 * *x*, *x* = follow-up months).

Among asymptomatic patients, it has been shown that patients with drug-induced Type 1 ECG had a better prognosis than patients with spontaneous Type 1 ECG. The occurrence of lethal events in patients with spontaneous Type 1 ECG was 0.5%–1.2%/year, and that in patients with drug-induced Type 1 ECG was 0%–0.4%/year.^56,69,76,80–82^ The risk of VF in asymptomatic patients with spontaneous Type 1 ECG has been reported to be 3.9–5.3 times higher than that in asymptomatic patients with drug-induced Type 1 ECG.^56,69,76,80–82^  *Figure 3A* shows a bubble plot of prognosis in asymptomatic patients with and without spontaneous Type 1 ECG. Patients with drug-induced Type 1 ECG had better prognosis (0.29%/year) than patients with spontaneous Type 1 ECG (0.88%/year).

Among patients who had already experienced syncopal episodes, patients with drug-induced Type 1 ECG also had a better prognosis (i.e. VF episodes) than patients with a spontaneous Type 1 ECG (0.67%–1.9%/year vs. 0.82–3.2%/year, respectively). The risk of VF in patients with spontaneous Type 1 ECG was 1.2–6.4 times higher than that in patients with drug-induced Type 1 ECG.^56,73,81,83,88^ This prognostic value is also true in patients without previous cardiac arrest.^89–92^

In patients who already had VF or aborted cardiac arrest, the risk for future VF events was the same in patients with and those without spontaneous Type 1 ECG. The occurrence of VF in patients with spontaneous Type 1 ECG was 7.4%–10.2%/year, and that in patients with drug-induced Type 1 ECG was 3.4%–10.6%/year.^77,87,93^ Some studies combined patients with syncope and patients with VF as symptomatic patients. *Figure 3B* shows prognosis of symptomatic patients who experienced syncope or VF. Event rates were 4.08%/year in symptomatic patients with spontaneous Type 1 ECG and 1.34%/year in symptomatic patients with drug-induced Type 1 ECG. In addition, the risk of VF recurrence in unexplained cardiac arrest survivors did not differ between those with drug-induced Type 1 ECG and those with a negative SCBT.^94^

Among patients with suspected BrS, such as patients having saddleback type ECG or family history, patients with negative response to SCBT have better prognosis than patients with positive response.^34,53,60,67,68^ Evian *et al.* showed that 3 out of 93 patients with positive response had ventricular tachyarrhythmic events, but 65 patients with negative response did not have the events among patients having baseline Type 2 or Type 3 ECG without previous VF episodes.^67^ Asymptomatic patients with negative response had excellent prognosis.^31,53^ Patients with syncope and negative response also had good prognosis, but attention should be paid if mechanism of syncope is arrhythmogenic.^68^ Type 2 ECG at baseline is more frequently observed in patients with positive response than patients with negative response.^53,68^ Negative response in patients with baseline Type 2 ECG could be occurred by false-negative response^16,68,95^ or incomplete right bundle branch block with ST elevation that resembles saddleback morphology.^96^

Children and young patients with drug-induced Type 1 ECG also have a lower risk for VF than patients with spontaneous Type 1 ECG.^97–99^ Spontaneous Type 1 ECG is associated with VF events.^97–100^ However, spontaneous Type 1 ECG is not associated with recurrent arrhythmic events in young patients who have already experienced ventricular tachyarrhythmic events.^101^

Spontaneous Type 1 ECG could not predict prognosis in women.^102–104^ Sieira *et al.* reported prognosis in 228 women with BrS.^103^ Spontaneous Type 1 ECG was less frequent in women (7.9%) than that in men (23.2%). Aborted cardiac arrest, SND, and atrial fibrillation could predict occurrence of VF in women, whereas spontaneous Type 1 ECG was not associated with VF events. Bethome *et al.* and Rodriguez-Manero *et al.* also showed that spontaneous Type 1 is less frequent in women than that in men, and it was not a predictor of prognosis in women.^102,104^

Patients with *SCN5A* mutation frequently have spontaneous Type 1 ECG than patients without *SCN5A* mutation.^105^ There were two meta-analysis that showed prognostic significance of ECG types in patients with and without *SCN5A* mutation; however, the data were analyzed from a limited number of patients in both studies. Yang *et al.* reported that ECG type did not affect prognosis in patients with and without *SCN5A* mutation.^106^ Rattanawong *et al.* showed that major arrhythmic events more increased in patients with *SCN5A* mutation and spontaneous Type 1 ECG than did in patients with SCN5A mutation and drug-induced Type 1 ECG.^107^

In addition to the signature ST elevation pattern, various other ECG changes have been reported after a SCB challenge. The ECG changes include PQ and QRS interval prolongation,^12,43,44,50,108–110^ left axis deviation,^111^ peripheral Type 1 ECG,^74,112–114^ VAs,^16,34,42–45,48,50,56,60^ (*see previous paragraph*), and elimination of J wave.^115^ Some of the ECG changes might be associated with future VF events. The studies on peripheral Type 1 ECG included both spontaneous and drug-induced Type 1 ECGs.^74,112–114^ Peripheral Type 1 ECG was observed in 9%–20% of patients with ajmaline-positive test and in 10% of patients with spontaneous Type 1 ECG, and it was also frequently observed in patients with *SCN5A* mutation. The risk for VF is 2.3–5.0 times higher in patients with peripheral Type 1 ECG than that in patients without peripheral Type 1 ECG.

Ventricular arrhythmias can occur during SCBT (SCBT-induced VAs; *see previous paragraph*). There have been only a few studies in which relations between SCBT-induced VAs and prognosis were evaluated,^34,45,49^ and the prognostic value of SCBT-induced VAs is controversial. The incidences of SCBT-induced VAs might differ depending on the kind of SCB used, and SCBT-induced VAs can occur in patients who did not show Type 1 ECG after administration of SCB.^112^ An early study including 23 patients in whom pilsicainide test was performed and who were followed for 45 months showed no association between SCBT-induced VAs and prognosis.^49^ Conte *et al.* reported that ajmaline-induced life-threatening VAs occurred in 9 (1.8%) of 503 patients with drug-induced Type 1 ECG. Ventricular fibrillation or sudden cardiac death did not occur in any of the patients with ajmaline-induced VAs during a 29-month follow-up period.^45^ Ueoka *et al.* reported that SCBT-induced VAs could predict VF events in 245 patients including 181 patients with spontaneous Type 1 ECG.^34^ In that study, pilsicainide induced VAs in 24 patients (9.8%). The incidence of VF events during a 113-month follow-up period was 7.1%/year in patients with pilsicainide-induced VAs, whereas it was only 0.89%/year in patients without pilsicainide-induced VAs. The risk of VF was approximately seven times higher in patients with SCBT-induced VAs. Although these data might indicate a role for SCBT in predicting risk in patients with spontaneous Type 1 ECG, the 2022 ESC guideline does not recommend SCBT in these patients because of the risk of VF.^116^

### Considerations and conclusions

The SCBT is widely used to unmask a Brugada pattern in the right precordial leads. Studying specificity and sensitivity of the SCBT is difficult because there is no real “gold standard.” Furthermore, not all drugs (procainamide, flecainide, ajmaline, and, in particular in Japan, pilsicainide) have been equally well studied. Given the importance of the RVOT epicardial substrate in arrhythmogenesis, it would be of interest to study the presence of the substrate in relation to the result of the test in more detail, but this has, as far as we know, not be done.

Based on all available evidence, we do believe that specificity of the test is far from perfect which means that a significant number of patients with a positive test might not have BrS. To the contrary, sensitivity of the test seems descent, certainly for ajmaline, which means that a negative test indeed means that BrS is highly unlikely.

**Figure 4:**
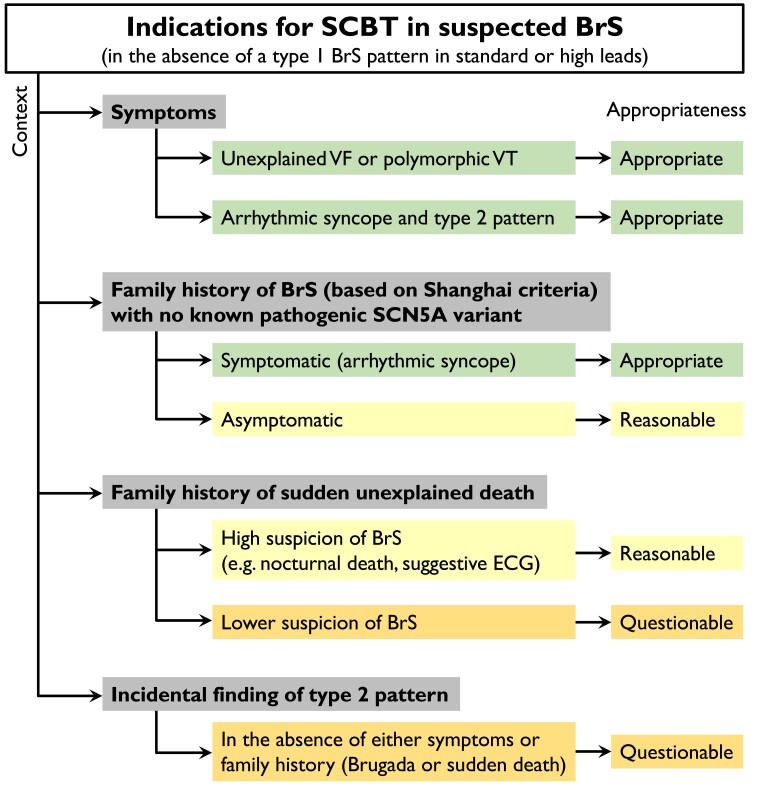
Appropriateness of sodium channel blocker testing in common clinical scenarios. These appropriateness criteria for sodium channel blocker test are the authors’ expert opinion based on concerns regarding sodium channel blocker test specificity and the generally excellent prognosis of drug-induced Brugada syndrome in asymptomatic individuals, as discussed in text. BrS, Brugada syndrome; SCBT, sodium channel blocker test; VF, ventricular fibrillation; VT, ventricular tachycardia.

Because of the debated specificity and the overall very low risk for future events in asymptomatic individuals, patients should be properly selected and counseled before SCBT is performed (*Figure 4*). In our expert opinion, an SCBT test can be considered appropriate in patients with documented VF or polymorphic VT that remains unexplained following extensive clinical assessment (including coronary angiography, cardiac magnetic resonance imaging, and exercise testing). Similarly, the test is appropriate in patients with syncope of likely arrhythmic etiology in the presence of a Type 2 Brugada ECG and in symptomatic first-degree relatives of a patient with a definite diagnosis of BrS based on Shanghai criteria and no known pathogenic or likely pathogenic *SCN5A* variant identified in the family (*Figure 4*). The test can be considered reasonable in an asymptomatic first-degree relative of a patient with a definite diagnosis of BrS based on Shanghai criteria and no known pathogenic or likely pathogenic *SCN5A* variant identified in the family, as well as in relatives of a patient with a diagnosis of BrS or conduction disease in presence of a rare variant of unknown significance in *SCN5A* in conjunction with genetic testing for co-segregation analysis. A SCBT is also reasonable in a first-degree relative of a sudden unexplained death victim in presence of high suspicion for BrS, such as nocturnal death in multiple individuals, and suggestive ECGs in surviving relatives or in the deceased prior to death. In a study from the UK challenging all first-degree relatives of a sudden arrhythmic death syndrome victim (regardless the presence of a high suspicion on BrS), the yield was relatively high (almost 30%),^117^ but given the unknown, but certainly not perfect specificity of the SCBT, we prefer to limit the test to those with high suspicion on BrS. In all others, we consider the test questionable. The test should also be considered questionable in individuals with an incidental finding of a Type 2 Brugada ECG in the absence of arrhythmic syncope, cardiac arrest, and family history of BrS or sudden unexplained death. Finally, SCBT should be considered inappropriate for screening individuals in the absence of a Type 2 Brugada ECG, family history of BrS or sudden death, or personal history of arrhythmic syncope or cardiac arrest, and it is not recommended in all patients with a prior Type 1 Brugada pattern.^116^ A potential thought is that, alike the presence of early repolarization, a positive SCBT indicates the presence of an arrhythmic substrate reflecting low RVOT conduction reserve which may turn out to be a risk factor for VA in the future. Indeed, in a large Finnish cohort of middle-aged individuals, the presence of early repolarization in the inferior leads was shown to convey a risk for sudden death 20–25 years later, potentially associated with an acute myocardial infarction.^118^ This association could be established because large cohorts with baseline ECGs and long follow-up are available. Such cohorts are not available for patients with a positive SCBT for the time being. Establishing such an association between the drug-induced Type I BrS ECG and long-term arrhythmic events would confirm the prognostic utility of the test. After all, the objective of screening asymptomatic individuals is the prediction of major events and mitigation of such risk, not only establishing an ECG diagnosis (*Graphical Abstract*).

## Data Availability

No data were generated or analyzed for or in support of this paper.
